# Supplementary material for: Predation and fragmentation portrayed in the statistical structure of prey time series
Source: BMC Ecol. 2009 May 6;9:10. doi: 10.1186/1472-6785-9-10 (PMC2689204; doi:10.1186/1472-6785-9-10)
Supplement: Additional file 2 — Voles and related classes ODDox Documentation. ODDox documentation of the agent-based model (ALMaSS) applied by Hendrichsen et al. The documentation is started by activating main.html. [file 1472-6785-9-10-S2.zip › Vole_ODDox/globals_0x73.html]

ALMaSS ODDox: Class Members

- Main Page
- Related Pages
- Classes
- Files

- File List
- File Members

- All
- Functions
- Variables
- Typedefs
- Enumerations
- Enumerator
- Defines

- \_
- a
- b
- c
- d
- f
- g
- h
- i
- l
- m
- n
- o
- p
- r
- s
- t
- u
- v
- w

Here is a list of all file members with links to the files they belong to:

### - s -

- sa\_cattle\_is\_out
  : setaside.h- sa\_cattle\_out
    : setaside.h- sa\_cut\_to\_hay
      : setaside.h- sa\_cut\_to\_silage
        : setaside.h- sa\_glyphosate
          : setaside.h- sa\_start
            : setaside.h- sa\_wait
              : setaside.h- SETASIDE\_BASE
                : setaside.h- SetAsideToDo
                  : setaside.h- sleep\_all\_day
                    : treatment.h- spring\_harrow
                      : treatment.h- spring\_plough
                        : treatment.h- spring\_roll
                          : treatment.h- spring\_sow
                            : treatment.h- start
                              : treatment.h- straw\_chopping
                                : treatment.h- strigling
                                  : treatment.h- strigling\_sow
                                    : treatment.h- stubble\_harrowing
                                      : treatment.h- swathing
                                        : treatment.h- syninsecticide\_treat
                                          : treatment.h

---

Generated on Thu Jan 22 14:13:48 2009 for ALMaSS ODDox by 
 1.5.6
